# Effect of Ga Doping on the Stability and Optoelectronic Properties of ZnSnO Thin Film Transistor

**DOI:** 10.3390/mi15121445

**Published:** 2024-11-29

**Authors:** Liang Guo, Qing Wang, Chao Wang, Xuefeng Chu, Yunpeng Hao, Yaodan Chi, Xiaotian Yang

**Affiliations:** 1 Key Laboratory of Architectural Cold Climate Energy Management, Ministry of Education, Jilin Jianzhu University, Changchun 130118, China; guoliang@jlju.edu.cn (L.G.); wq785020874@163.com (Q.W.); stone2009@126.com (X.C.); apeng0031@163.com (Y.H.); chiyaodan@jlju.edu.cn (Y.C.); 2 Department of Basic Science, Jilin Jianzhu University, Changchun 130118, China; 3 School of Electrical and Computer Science, Jilin Jianzhu University, Changchun 130118, China; 4 Department of Chemistry, Jilin Normal University, Siping 136000, China

**Keywords:** thin film transistor, co-sputtering, electrical properties, stability, ultraviolet photovoltaics

## Abstract

The electrical, stability and optoelectronic properties of GZTO TFTs with different Ga doping concentrations were investigated. Active layers were prepared by co-sputtering GaO and ZTO targets with different sputtering powers. The experimental results show that the surface of GZTO films is smooth, which is favorable for stability. The off-state current is reduced by a factor of 10, the switching ratio is increased to 1.59 × 10^8^, and the threshold voltage shift is reduced in PBS and NBS tests. In addition, the transmittance of all devices is greater than 80% in the visible range, and the optical bandgap of the TFTs is increased from 3.61 eV to 3.84 eV after Ga doping. The current enhancement of the GZTO TFTs is more pronounced under UV irradiation, with higher responsiveness and better-sustained photoconductivity. It is proved that Ga doped into ZTO as a carrier suppressor can better combine with oxygen vacancies and reduce the concentration of oxygen vacancies and oxygen defects compared with Zn and Sn atoms, thus improving stability. GaO, as a wide bandgap material, can improve the optical bandgap of GZTO TFTs so that they can better absorb the light in the UV wavelength band, and they can be used in the field of UV photodetection.

## 1. Introduction

In recent years, amorphous metal–oxide–semiconductor (AOS) thin-film transistors (TFTs) have become a research hotspot in the field of display due to their excellent characteristics such as high mobility, excellent uniformity, and high visible transmittance, which are suitable for advanced large-area electronic devices and are also one of the most promising devices for high-end integrated circuits [1,2]. Indium-containing metal oxide semiconductors, such as InZnO, InGaZnO and HfInZnO, have been widely studied due to the high carrier mobility generated by the special electronic configuration of the In ions (n−1)d^10^ns^0^, and in particular, IGZO TFTs have been applied to flat panel displays (FPDs) [3,4,5]. Although In-based AOSs show good performance, their fabrication cost and safety will hinder their long-term development in TFTs since In is an expensive and toxic material. In-free oxide semiconductor materials were developed for the fabrication of low-cost, safe, and effective TFTs [6]. In contrast, Sn not only has an electronic structure similar to that of In, but is also non-toxic and less costly. Zn-Sn-O (ZTO) is considered a prominent candidate for In-free amorphous semiconductor materials. With the advantages of low cost, non-toxicity, high mobility, and good transparency, the ZTO film became a promising candidate for transparent thin-film transistor channels. Second, ZTO films are prepared using a low-temperature process, which makes it easy to fabricate thin-film transistors and circuits on flexible substrates. Furthermore, it has been shown that the structure of ZTO films gradually changes from polycrystalline to amorphous as the proportion of Sn^4+^ increases, while the film surface becomes more uniform and denser [7,8]. However, ZTO films have difficulty obtaining excellent switching characteristics and bias stability due to the high carrier concentration in the absence of gallium, which acts as a carrier suppressor in oxide semiconductors. Gallium can combine with oxygen more easily and reduce the oxygen vacancies in oxide semiconductor films, thereby inhibiting the formation of defects. In addition, GaO materials have a property that makes them better suited for use in photodetection. Gallium oxide (GaO), as a new ultra-wideband semiconductor material, has a number of significant advantages, with a bandgap in the range of 4.8–5.3 eV, with a wider bandgap GaO doping makes the entire bandgap of the GZTO film has been improved, the wider the bandgap, the absorption of shorter wavelengths of ultraviolet light produced by the more pronounced photoelectric effect, which is its role in the field of optoelectronic detection is better! The wider the band gap, the more obvious the photoelectric effect produced by its absorption of shorter wavelengths of ultraviolet light, which is its better role in the field of photoelectric detection. This property gives gallium oxide higher power characteristics and deep UV photovoltaic properties compared to other semiconductor materials. There are very few device studies on GZTO materials, and in this paper, we will study and analyze their electrical properties, stability, and UV photovoltaic characteristics [9,10].

## 2. Experimental

**Figure 1:**
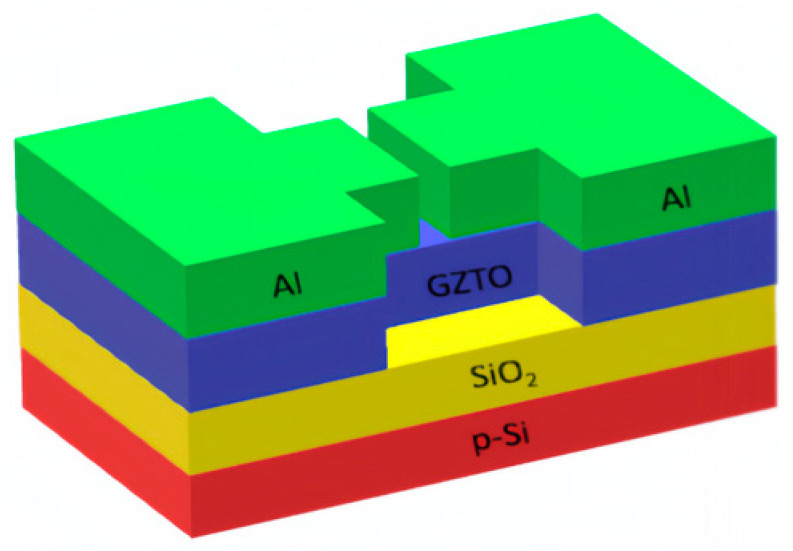
Structure of GZTO TFTs.

The main structure of a TFT device is shown in Figure 1 and includes a substrate, an insulating layer, an active layer, and a source-drain. First, p-Si was used as the substrate and gate, and thermally oxidized 285 nm thick SiO_2_ was used as the insulating layer, which was cleaned in an ultrasonic cleaner using acetone, anhydrous ethanol, and deionized water. The active layer was deposited by magnetron sputtering after processing the lithography mask with a chamber pressure of 8 × 10^−4^ torr, an Ar_2_ to O_2_ ratio of 90:10, and a channel length of 10 μm and a width of 300 μm. The active layer was co-sputtered at room temperature using a ZTO (Zn:Sn = 5:5) target with a sputtering power of 80 W and different sputtering powers of 0 W, 15 W, 30 W, 45 W, and 60 W. The GaO target was co-sputtered using a ZTO target with a sputtering power of 80 W and different sputtering powers of 0 W, 15 W, 30 W, 45 W, and 60 W. GaO targets with different sputtering powers of 0 W, 15 W, 30 W, 45 W, 60 W were co-sputtered and the samples were denoted as ZTO, GZTO-15, GZTO-30, GZTO-45, GZTO-60, respectively. The mask was subsequently removed and rapidly annealed under air atmosphere using 300 °C for 1 h. Finally, 50 nm-thick Al was evaporated using an e-beam to serve as the source-drain electrodes.

The morphologies of the films were tested by Oxford MFP-3D Atomic Force Microscope (AFM, Santa Barbara, CA, USA), and the transmittance of the film was measured by UV-2600 UV-visible spectrophotometer (Tokyo, Japan). X-ray photoelectron spectroscopy (XPS) measurements were performed using Thermo Fisher Scientific ESCALAB 250Xi (Waltham, MA, USA) and photoelectron spectroscopy system, The electrical characteristics of ZTO thin-film transistors were measured by Keysight B1500A, semiconductor parameter meter (Santa Rosa, CA, USA). Light response testing was performed using a 405 nm laser lamp with a power density of 64 μm/cm^2^ and manual masking to achieve light pulses.

## 3. Results and Discussion

### 3.1. Thin Film Properties

**Figure 2:**
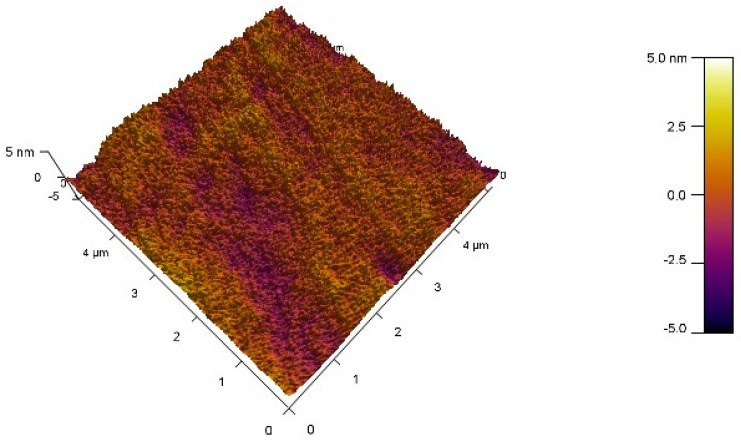
AFM surface topography of thin films.

Figure 2 shows the surface morphology of GZTO-15 measured by AFM, which is used to characterize the roughness and surface morphology of the film. Table 1 shows the AFM test plots of GZTO films with different gallium doping concentrations. Table 1 lists the RMS values of different films. The results show that the roughness of the annealed films is slightly reduced compared to the unannealed films because the moderate increase in annealing temperature causes the atoms to rearrange and the structure of the film is adjusted, which helps to reduce the defects in the film and thus improves the quality of the film [11]. The annealing does not have a significant effect on the roughness of the films with different Ga doping concentrations, which are all below 0.8 nm, indicating that the films prepared by magnetron sputtering using the co-sputtering method have a smooth and homogeneous surface, Low roughness means that the surface of the film is flatter, which facilitates the orderly growth of molecules in the active layer. Films with low roughness form a good contact surface with the gate dielectric layer or other neighboring layers. This good contact helps in carrier migration and improves the overall performance of the device. It helps reduce defects and pores in the film. Dense films provide better barrier properties against penetration of external substances such as gases and water molecules, thereby extending the life of the device. In thin-film transistors, low-roughness active layer films can reduce charge traps and leakage currents caused by surface roughness, thereby improving device stability and reliability [12].

**Figure 3:**
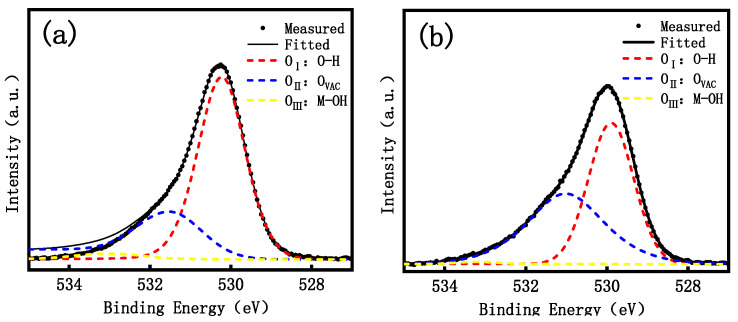

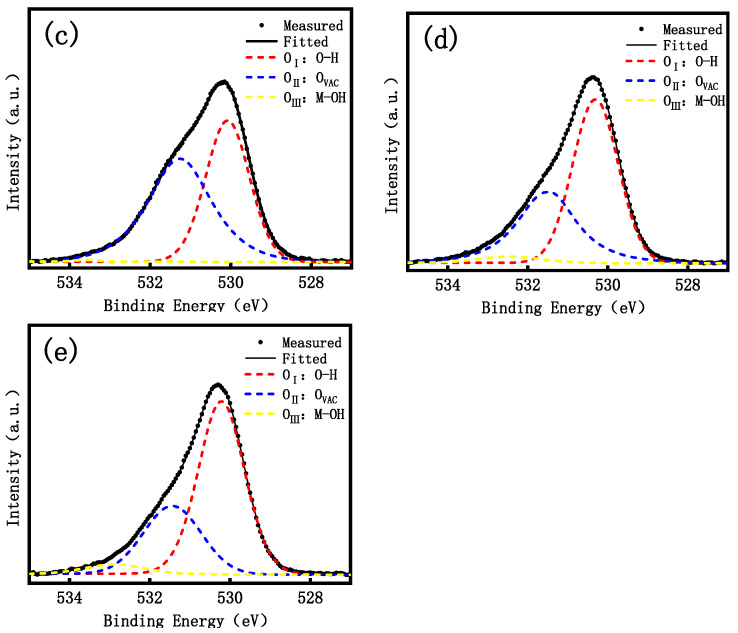
XPS test analysis of thin films (**a**) ZTO; (**b**) GZTO-15; (**c**) GZTO-30; (**d**) GZTO-45; (**e**) GZTO-60.

**Figure 4:**
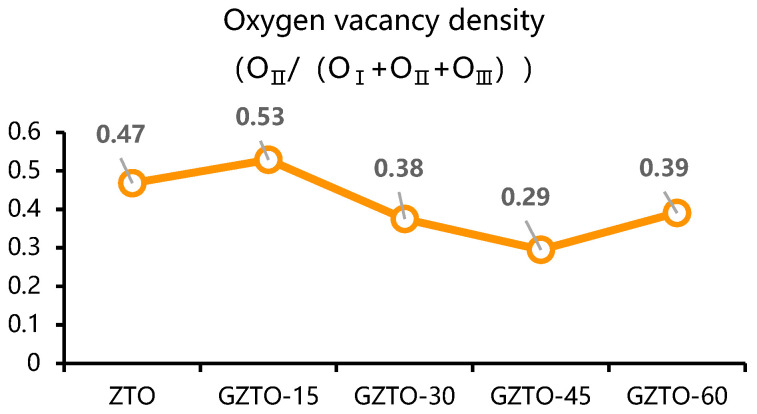
Oxygen vacancy density (O_II_/O_I_ + O_II_ + O_III_).

Table 2 lists the elemental composition of each thin film transistor. In order to observe more deeply the effect of different Ga doping concentrations on the internal chemical composition of GZTO films, they were analyzed by XPS O1s, and the experimental results are shown in Figure 3. The O_1s_ peaks in the figure were superimposed and fitted by the Gaussian distribution method and divided into three sub-peaks, O_I_, O_II_, and O_III_, which are centered at 530.1 eV, 531.6 eV, and 532.8 eV. Among them, O_I_ and O_II_ are associated with O^2−^, the M-O bond is considered to be produced by lattice oxygen O^2−^ ions in the oxide film, OVAC is produced by oxygen vacancy O^2−^ ions in the oxide film, and O_III_, including O-H and C-O groups, is associated with impurities. O_II_/(O_I_, O_II_, O_III_) denotes the density of the oxygen vacancies in the film, which is shown in Figure 4, since the GaO and ZTO co-sputtering is used, the low concentration of Ga doping leads to a slight increase in the oxygen vacancies, this is due to the substitution of Ga atoms for Zn atoms in the ZnO lattice, leading to lattice distortion and increased oxygen defects. This value decreases from 52.9% to 29.5% with the increase of Ga concentration, which proves that the doping of Ga can reduce the concentration of oxygen vacancies in the film. It is well known that in the active layer of thin-film transistors, oxygen vacancies can either be used as carriers to increase the carrier concentration or as traps to increase the scattering effect during carrier transport, thus decreasing the carrier concentration, which is very much correlated with the electrical performance and stability of thin-film transistors. Compared to Zn and Sn atoms, Ga atoms have a stronger ability to bind to oxygen vacancies, so Ga atoms can create charge compensation in crystals, which reduces the oxygen vacancies and leads to an improvement in the voltage shift phenomenon of the transistor [13,14].

**Figure 5:**
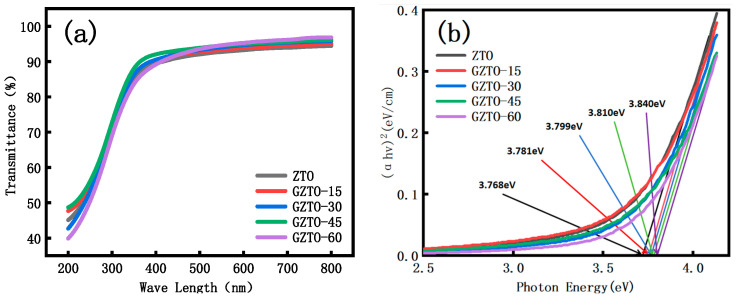
(**a**) Transmittance; (**b**) Absorbance and optical band gap.

GZTO was deposited on a sapphire substrate and tested for transmittance and absorbance. As shown in Figure 5a, the transmittance spectra are in the wavelength of 200–800 nm, and the transmittance is higher than 90% in the visible range, which proves that it can be used in the field of transparent display [15]. Figure 5b shows the absorbance and optical band gap of GZTO films with different Ga doping concentrations. Compared with the ZTO film, the optical band gap of the GZTO film is improved, which is due to the higher optical band gap of GaO material, and the GZTO co-sputtered with ZTO also has a higher optical band gap. In addition, from the microscopic level, the interaction of doped Ga atoms with Zn and Sn atoms in the film changes the energy level structure and enters the Fermi energy level of the valence and conduction bands, so that the energy states at the top of the low-conductivity or valence bands have been occupied, which leads to the enlargement of the optical bandgap [16]. The phenomenon of a larger optical band gap occurs, a phenomenon known as the Burstein–Moss effect. The shorter wavelength and higher photon energy of UV light can meet the energy required for high optical band gap materials to jump from the valence band to the conduction band. In summary, it can be seen that GZTO TFT with a higher optical bandgap can absorb higher energy UV light with higher efficiency than other materials and excite the electrons in the material, so that it has good photoelectric performance under UV irradiation, which can be applied to photodetectors and solar cells [17,18,19].

### 3.2. Electrical Performance

**Figure 6:**
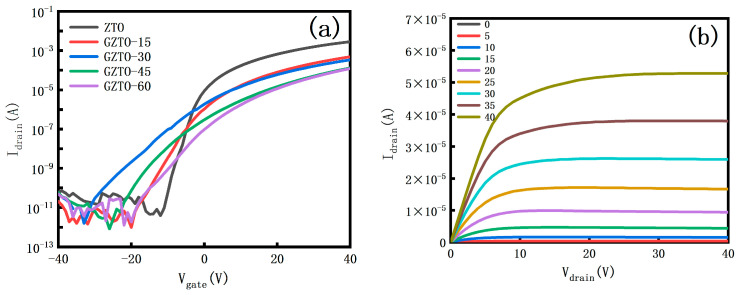
(**a**) Transfer characteristic curve; (**b**) Output characteristic curve.

From the transfer and output curves in Figure 6, it is observed that the transfer characteristics of the TFT are good, and with the increase of GaO sputtering power, the curve shows an overall downward trend to the right, and there is a significant reduction in the open-state current and the off-state current. From the output curve, it can be seen that the TFT has a cutoff region, a linear region, and a saturated region, and the I_D_ increases with the increase of the V_D_, which is typical of the N-channel TFT. No current crowding occurs when the V_DS_ is small phenomenon, indicating that the source-drain electrode is in good contact with the active layer [20,21].

From the data in Table 3, the doping of Ga makes the mobility of the TFT greatly reduced. The mobility tends to be positively correlated with the carrier concentration. Combined with the previous XPS analysis, gallium as a carrier inhibitor, in the annealing process, the binding energy of the Ga-O bond is greater than that of the Zn-O bond and the Sn-O bond, and combines with the oxygen diffused in the air, which reduces the oxygen vacancies. The intrinsic carrier concentration of the active layer decreases, and with the increase of the doping concentration, the GZTO mobility gradually decreases. Associated with this are the open-state current and the off-state current, which also decrease with decreasing carrier concentration. This also results in the need for a higher gate voltage to sense more carriers to turn the device on, and the threshold voltage also increases with increasing doping concentration.

### 3.3. Stability

**Figure 7:**
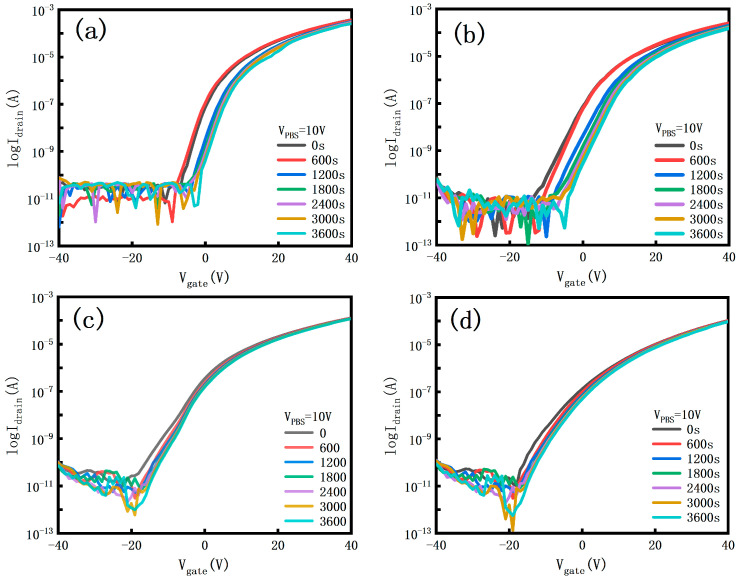

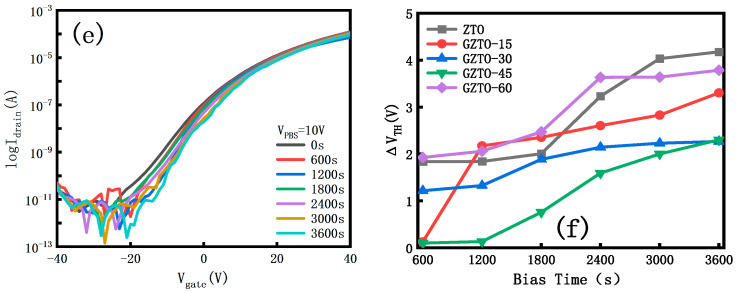
Device transfer curves under forward bias stresses (**a**) ZTO; (**b**) GZTO-15; (**c**) GZTO-30; (**d**) GZTO-45; (**e**) GZTO-60; (**f**) Threshold voltage shift.

Bias stress stability is one of the key factors in measuring whether a device can be produced and applied in practice. Figure 7 demonstrates the positive gate bias stress stability (PBS) of all samples with a bias voltage of 10 V and a bias time of 3600 s. From the figure, it can be seen that a small amount of doped Ga element improves the overall stability of the device as compared to ZTO TFTs, but the threshold voltage shift is still larger as compared to GZTOs with a higher Ga doping concentration, which is attributed to the fact that GaO carries a large number of oxygen ions during sputtering, resulting in a rise in the number of oxygen vacancies and the number of oxygen defects rise, which reduces the device stability. With the increase in the doping concentration, GZTO-30 and GZTO-45 show better stability of the positive gate bias stress, which may be attributed to the larger binding energy of gallium atoms and oxygen atoms, which forms a more stable Ga-O bond. The reduction of oxygen vacancies and oxygen defect density reduces the scattering and trapping effects of carriers in the transport process, which improves the overall stability of the device; whereas the stability of GZTO-45 decreases, and the high doping concentration leads to the excess gallium atoms gathering at the grain boundaries, which hinders the growth of the grains, resulting in the inhomogeneity of the grain size [22,23].

**Figure 8:**
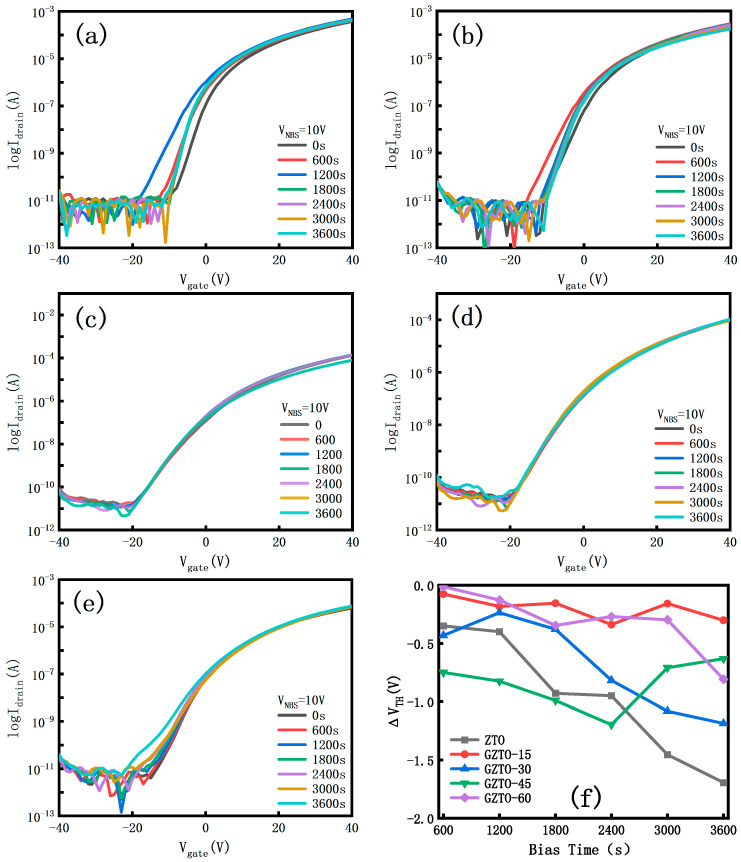
Device transfer curves under negative bias stresses (**a**) ZTO; (**b**) GZTO-15; (**c**) GZTO-30; (**d**) GZTO-45; (**e**) GZTO-60; (**f**) Threshold voltage shift.

Figure 8 shows the negative gate bias stress stability (NBS) for all samples at a bias voltage of −10 V and a bias time of 3600 s. The NBS results are similar to the PBS results, except that the absolute value of the threshold voltage shift in NBS is smaller than that in PBS. The shift curves and threshold voltage trends are similar to those of the PBS test results, except that the absolute value of the threshold voltage shift in NBS is smaller than that in PBS, which is typical of N-type semiconductors because carrier trapping is more important as a multiplet than hole trapping under negative bias conditions [24].

### 3.4. UV Photoelectric Properties

**Figure 9:**
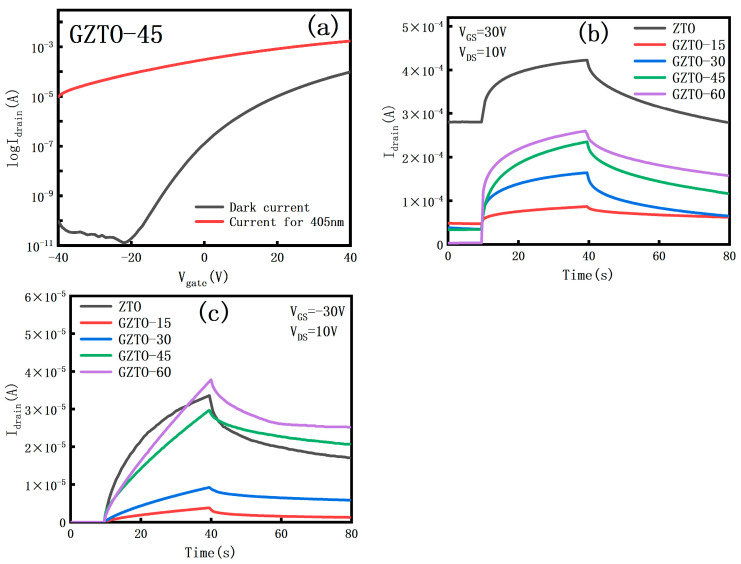
(**a**) Transfer curves measured under UV irradiation and in a dark environment; (**b**) I-t diagram under UV illumination with +30 V gate voltage; (**c**) I-t diagram under UV illumination with −30 V gate voltage.

Figure 9a demonstrates the transfer curves of the TFTs irradiated by UV light at a wavelength of 405 nm, and the leakage currents are all increased compared to those of the transfer curves in the dark environment [25]. The Figure 9b demonstrates the I-t diagram of the GZTO TFT irradiated by 405 nm UV light under V_GS_ = 30 V and V_DS_ = 10 V, and the I_total_/I_dark_ of the GZTO TFT is close to about 10^2^. Even a small amount of Ga doping makes GZTO much more responsive to light, ahead of ZTO TFT’s Ilight/Idark, which is only about two times more responsive to light [26,27,28]. (c) The figure demonstrates that the GZTO TFT is more responsive to light with slower recovery time and sustained photoconductivity under V_GS_ = −30 V and V_DS_ = 10 V; the more obvious the leakage current increases with the increase of doping concentration. This is due to the fact that when the UV light is irradiated, the energy of the incident photons is larger than the optical band gap of GZTO and is absorbed in the active layer, and the higher energy satisfies the electron to jump from the valence band to the conduction band, forming photogenerated charge carriers, and increasing the concentration of free electrons and holes. Thin-film transistors with high optical bandgap materials generally have lower electron concentration and higher resistivity, which would make them exhibit more significant photoelectric effect under UV irradiation [29,30].

## 4. Conclusions

As a result of this experiment, GZTOs prepared by co-sputtering of GaO and ZTO with different Ga doping concentrations were investigated and their film and device properties were analyzed. The results indicate that the films prepared by magnetron sputtering using the co-sputtering method all have smooth and uniform surfaces with reduced defects, which is favorable to the electrical properties of the thin film transistors composed of them. Ga doping also reduces the oxygen vacancy density in the active layer, and the higher bonding energy of the Ga-O bonding improves the stability of the devices. The GZTO films all have good transmittance, and their optical bandgaps are improved with the increase of Ga. The optical band gap of GZTO films is also improved with the increase of Ga doping concentration; in terms of electrical performance, the carrier concentration of GZTO devices is suppressed, so the mobility and off-state current are decreased, and the current-switching ratio is improved, which enhances the stability of the devices. In the bias test, it is confirmed that all devices can still obtain better stability under longer bias voltage, and the GZTO-45 device can obtain higher bias voltage stability, and the threshold voltage shift phenomenon has been improved. Finally, by comparing the transfer curves of UV irradiation and dark environment tests and the I-t diagrams of GZTO TFTs under UV irradiation with different gallium doping concentrations, it is proved that the Ga-doped GZTO devices with higher optical bandgap have a more sensitive response to UV light, and this finding suggests that the TFTs with GZTO as the active layer can be applied to the field of UV photodetectors.

## Figures and Tables

**Table 1:** Thin film surface roughness.

TFT	RMS (nm)
GZTO TFT-unannealed	0.94
ZTO TFT 300 °C	0.71
GZTO-15 TFT 300 °C	0.62
GZTO-30 TFT 300 °C	0.55
GZTO-45 TFT 300 °C	0.66
GZTO-60 TFT 300 °C	0.79

**Table 2:** Thin film element composition.

TFT	Ga3d (%)	Zn2p (%)	Sn3d (%)	O1s (%)
ZTO	0	19.17	18.04	62.79
GZTO-15	1.08	18.62	16.33	63.99
GZTO-30	1.72	17.42	17.88	62.98
GZTO-45	2.22	16.33	17.95	63.48
GZTO-60	2.81	18.97	17.00	61.21

**Table 3:** TFT Electrical Performance Data.

TFT	μ_FE_ (cm^2^/vs)	V_TH_ (V)	SS (V/dec)	I_ON_ (A)	I_OFF_ (A)	I_ON_/I_OFF_
ZTO	10.82	1.82	2.31	2.84 × 10^−3^	5.63 × 10^−11^	5.04 × 10^7^
GZTO-15	2.80	9.19	3.08	4.83 × 10^−4^	5.86 × 10^−12^	8.25× 10^7^
GZTO-30	1.92	8.93	2.15	3.37 × 10^−4^	1.59 × 10^−12^	2.12 × 10^8^
GZTO-45	1.24	15.93	1.99	1.31 × 10^−4^	8.27 × 10^−13^	1.59 × 10^8^
GZTO-60	1.16	16.09	2.14	1.21 × 10^−4^	1.28 × 10^−12^	9.43 × 10^7^

## Data Availability

The data that support the findings of this study are available from the corresponding authors upon reasonable request.
